# Gold nanoparticle based double-labeling of melanoma extracellular vesicles to determine the specificity of uptake by cells and preferential accumulation in small metastatic lung tumors

**DOI:** 10.1186/s12951-020-0573-0

**Published:** 2020-01-23

**Authors:** Pablo Lara, Sujey Palma-Florez, Edison Salas-Huenuleo, Iva Polakovicova, Simón Guerrero, Lorena Lobos-Gonzalez, America Campos, Luis Muñoz, Carla Jorquera-Cordero, Manuel Varas-Godoy, Jorge Cancino, Eloísa Arias, Jaime Villegas, Luis J. Cruz, Fernando Albericio, Eyleen Araya, Alejandro H. Corvalan, Andrew F. G. Quest, Marcelo J. Kogan

**Affiliations:** 1 Departamento de Química Farmacológica Y Toxicológica, Universidad de Chile, Santos Dumont 964, 8380494 Santiago, Chile; 2 Laboratory of Cellular Communication, Program of Cell and Molecular Biology, Center for Studies on Exercise, Metabolism and Cancer (CEMC), Institute of Biomedical Sciences (ICBM), University of Chile, Av. Independencia 1027, Santiago, Chile; 3 Advanced Center for Chronic Diseases (ACCDiS), Sergio Livingstone 1007, Santiago, Chile; 4 Centro de Medicina Regenerativa, Facultad de Medicina-Clinica Alemana, Universidad Del Desarrollo, Avenida las condes 12438, lo Barnechea, Santiago, Chile; 5 Laboratory of Oncology, Faculty of Medicine, Pontificia Universidad Católica de Chile, Portugal 61, Santiago, Chile; 6 Laboratorio de Análisis Por Activación Neutrónica, Comisión Chilena de Energía Nuclear, Nueva Bilbao, 12501 Santiago, Chile; 7 Centro de Biología Celular Y Biomedicina (CEBICEM), Facultad de Medicina Y Ciencia, Universidad San Sebastián, Lota 2465, Santiago, Chile; 8 Escuela de Medicina Veterinaria, Facultad de Ciencias de la Vida, Universidad Andrés Bello, Republica 440, Santiago, Chile; 9 Translational Nanobiomaterials and Imaging (TNI) Group, Radiology Department, Leiden University Medical Center, Albinusdreef 2, Leiden, The Netherlands; 10 CIBER-BBN, Networking Centre on Bioengineering, Biomaterials and Nanomedicine, and Department of Organic Chemistry, University of Barcelona, 08028 Barcelona, Spain; 11 Departamento de Ciencias Quimicas, Universidad Andres Bello, Republica 275, 8370146 Santiago, Chile; 12 Instituto de investigación Interdisciplinar en Ciencias biomédicas, Universidad SEK (I3CBSEK). Facultad Ciencias de La Salud, Fernando Manterola 0789, Santiago, Chile

**Keywords:** Extracellular vesicles, Exosomes, Gold nanoparticles, Metastasis, Tracking, Targeting, Drug delivery, Metastasis

## Abstract

**Background:**

Extracellular vesicles (EVs) have shown great potential for targeted therapy, as they have a natural ability to pass through biological barriers and, depending on their origin, can preferentially accumulate at defined sites, including tumors. Analyzing the potential of EVs to target specific cells remains challenging, considering the unspecific binding of lipophilic tracers to other proteins, the limitations of fluorescence for deep tissue imaging and the effect of external labeling strategies on their natural tropism. In this work, we determined the cell-type specific tropism of B16F10-EVs towards cancer cell and metastatic tumors by using fluorescence analysis and quantitative gold labeling measurements. Surface functionalization of plasmonic gold nanoparticles was used to promote indirect labeling of EVs without affecting size distribution, polydispersity, surface charge, protein markers, cell uptake or in vivo biodistribution. Double-labeled EVs with gold and fluorescent dyes were injected into animals developing metastatic lung nodules and analyzed by fluorescence/computer tomography imaging, quantitative neutron activation analysis and gold-enhanced optical microscopy.

**Results:**

We determined that B16F10 cells preferentially take up their own EVs, when compared with colon adenocarcinoma, macrophage and kidney cell-derived EVs. In addition, we were able to detect the preferential accumulation of B16F10 EVs in small metastatic tumors located in lungs when compared with the rest of the organs, as well as their precise distribution between tumor vessels, alveolus and tumor nodules by histological analysis. Finally, we observed that tumor EVs can be used as effective vectors to increase gold nanoparticle delivery towards metastatic nodules.

**Conclusions:**

Our findings provide a valuable tool to study the distribution and interaction of EVs in mice and a novel strategy to improve the targeting of gold nanoparticles to cancer cells and metastatic nodules by using the natural properties of malignant EVs.

## Introduction

Extracellular vesicles (EVs) secreted by different cell types [1–3] possess great potential for targeted therapy because they can transport cargos to specific sites in the body and protect their content from degradation [1–3]. Depending on their endogenous origin and composition, they can aid in preventing detection by the immune system, improve distribution and favor accumulation in specific tissues, an effect known as homing selectivity [4–11]. An example of the latter relates to cancer therapy, where some authors have proposed that tumor cells can capture more efficiently their own EVs in comparison with other cell-derived EVs, suggesting that tumor-specific proteins play essential roles in cellular uptake [12]. This property is interesting, as it could be exploited to develop delivery systems that enhance selectivity by preferentially targeting drugs to tumor cells.

Although the targeting of EVs towards tumors has been extensively studied, the assessment of biodistribution has mostly focused on subcutaneous xenograft models, which permit easier follow up because of their large size and the high degree of vascularization [13, 14]. Tracking EVs in metastasis can be challenging because of the small size of early tumors and the limitations of the light penetration, which is restricted to only a few millimiters. Lipophilic fluorescent dyes have been widely used to study the uptake and biodistribution of EVs as they can be easily incorporated into the membrane of the EVs and exhibit a wide range of excitation/emission spectra. The analysis of these probes is, however, limited by the potential transfer of these dyes to other extracellular components, and their aggregation, which leads to micelle formation and unspecific labeling of acceptor cells [15, 16]. Although the expression of stable fluorescent-proteins such as GFP or RFP seems to be an alternative to overcome these problems, their limited fluorescence intensity and light spectrum usually leads to low penetration and difficulties in their analysis in vivo [17]. Furthermore, fluorescence permits relative but not quantitative measurements, and is greatly affected by numerous external factors, including oxidation, scattering and bleaching [18–20].

Gold nanoparticles (AuNPs) are promising therapeutic agents, which have been widely studied in applications, such as drug delivery and diagnostics [21–26]. These nanoparticles are interesting for tracking analysis, as they display physical properties, such as surface plasmon resonance and scattering, which facilitates analysis by high-resolution imaging techniques, including computer tomography (CT), surface enhanced Raman spectroscopy (SERS), and photoacoustics [23, 27, 28]. Moreover, AuNPs possess the additional advantage of being highly biocompatible and more stable than fluorescence-based probes, allowing quantitative analysis deep inside the body [18–20, 29–31]. Although the encapsulation of AuNPs in extracellular vesicles has been reported previously, these studies focused primarily on the therapeutic application of gold and did not consider the effects of AuNP incorporation on the natural properties of the EVs, such as morphology, uptake or targeting. Labeling of the EVs with multiple AuNPs may represent a successful strategy to increase resolution for in vivo imaging [32]; however AuNP inclusion may also affect the density of the vesicles and therefore their accumulation and distribution. Direct labeling strategies that incorporate the AuNPs into the membrane of the EVs can affect their surface charge and block important ligands/receptors for the interaction with the acceptor cells. Indirect labeling-inclusion strategies can also alter cell viability and lead to contamination with apoptotic bodies and other extracellular components that can be isolated with the EVs [33]. Other methods, such as electroporation and sonication are well known to disrupt EV membranes and therefore alter their structure, composition as well as distribution [34]. While some attempts to resolve these problems have been reported [32, 35, 36], to the best of our knowledge, the development of a gold-labeling strategy which does not affect natural EV tropism, as well as the utilization of AuNPs for quantitative tracking of their accumulation/distribution in metastasis has not been described previously.

**Scheme 1.**
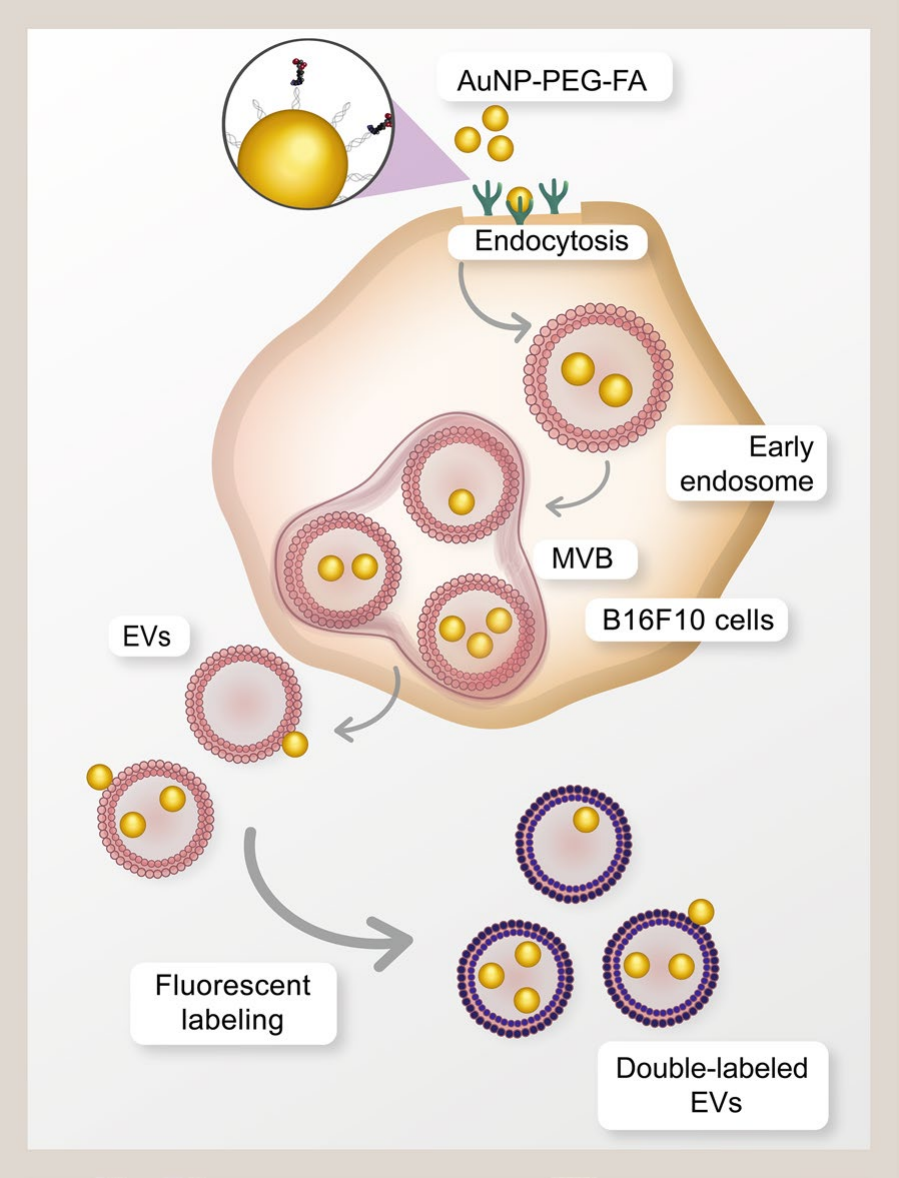
Proposed model to incorporate AuNP-PEG-FA and fluorescent dyes into B16F10 cell-derived EVs. MVB are multivesicular bodies

In this study, we defined the cell-type specific tropism of B16F10 melanoma-derived EVs and their targeting towards metastatic tumors by using fluorescence and gold-based analysis techniques. To this end, we established a novel protocol for the incorporation of AuNPs into EV preparations that involved the uptake of folic acid-conjugated AuNPs by B16F10 cells to promote nanoparticle internalization and trafficking through the late endosome pathway for subsequent release from cells in EVs. Our method did not affect the EV morphology, size distribution, surface charge, protein content, uptake or in vivo distribution when compared to unlabeled or fluorescently-labeled EVs. Fluorescence/computer tomography imaging, high sensitivity neutron activation analysis and gold-enhanced histological analysis were used to determine the distribution, quantitatively detect the accumulation, and determine the precise location of EVs in small metastatic tumors. With these approaches, we observed that B16F10 EVs preferentially accumulate in tumors and exploited this property as a strategy to increase AuNP delivery to metastatic nodules (Scheme 1).

## Results and discussion

### Isolation and characterization of EVs

**Fig. 1:**
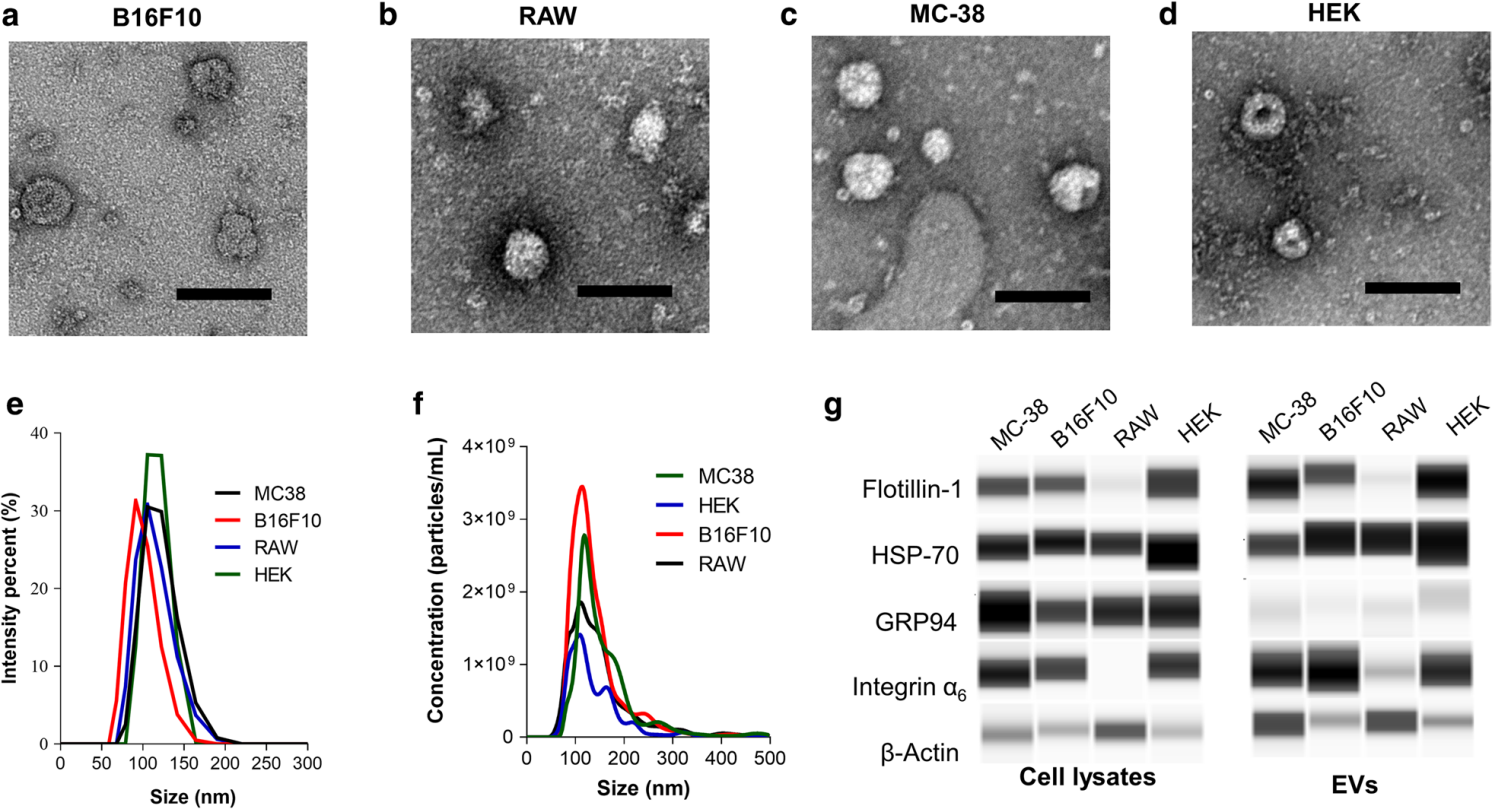
Characterization of cell-derived EVs. Representative TEM micrographs of EVs isolated from **a** B16F10, **b** RAW264, **c** MC-38 and **d** HEK293T cells. Scale bars are equivalent to 100 nm. **e** DLS and **f** NTA analysis of EVs. **g** Western blot of cell lysates and EVs

To stablish the preferential uptake of B16F10 cell-derived EVs, we isolated EVs from culture media of melanoma cells (B16F10), colon adenocarcinoma cells (MC-38), macrophages (RAW264), and embryonic kidney cells (HEK293T) under similar culture conditions. The resulting EVs were round in shape (Fig. 1a–d) with similar uniform size and vesicular-like shape, as described by other authors [12, 37, 38]. DLS and NTA analysis revealed similar hydrodynamic sizes, which averaged 115 nm, 118 nm, 113 nm and 128 nm for B16F10-EVs, MC-38-EVs, RAW264-EVs and HEK293T-EVs respectively (Fig. 1e, f). Western blotting was employed to detect some of the proteins commonly expressed in EVs. The markers, HSP70, Flotillin-1, Integrin α6, integrin β1 and β-actin were observed in all cell lysates and EV preparations, while the endoplasmic reticulum marker Grp94, used as negative control, was not detected in the EVs (Fig. 1g). Taken together, these observations are consistent with the notion that our preparations are highly enriched in small extracellular vesicles < 200 nm.

### Cell-type specific uptake of B16F10-EVs

**Fig. 2:**
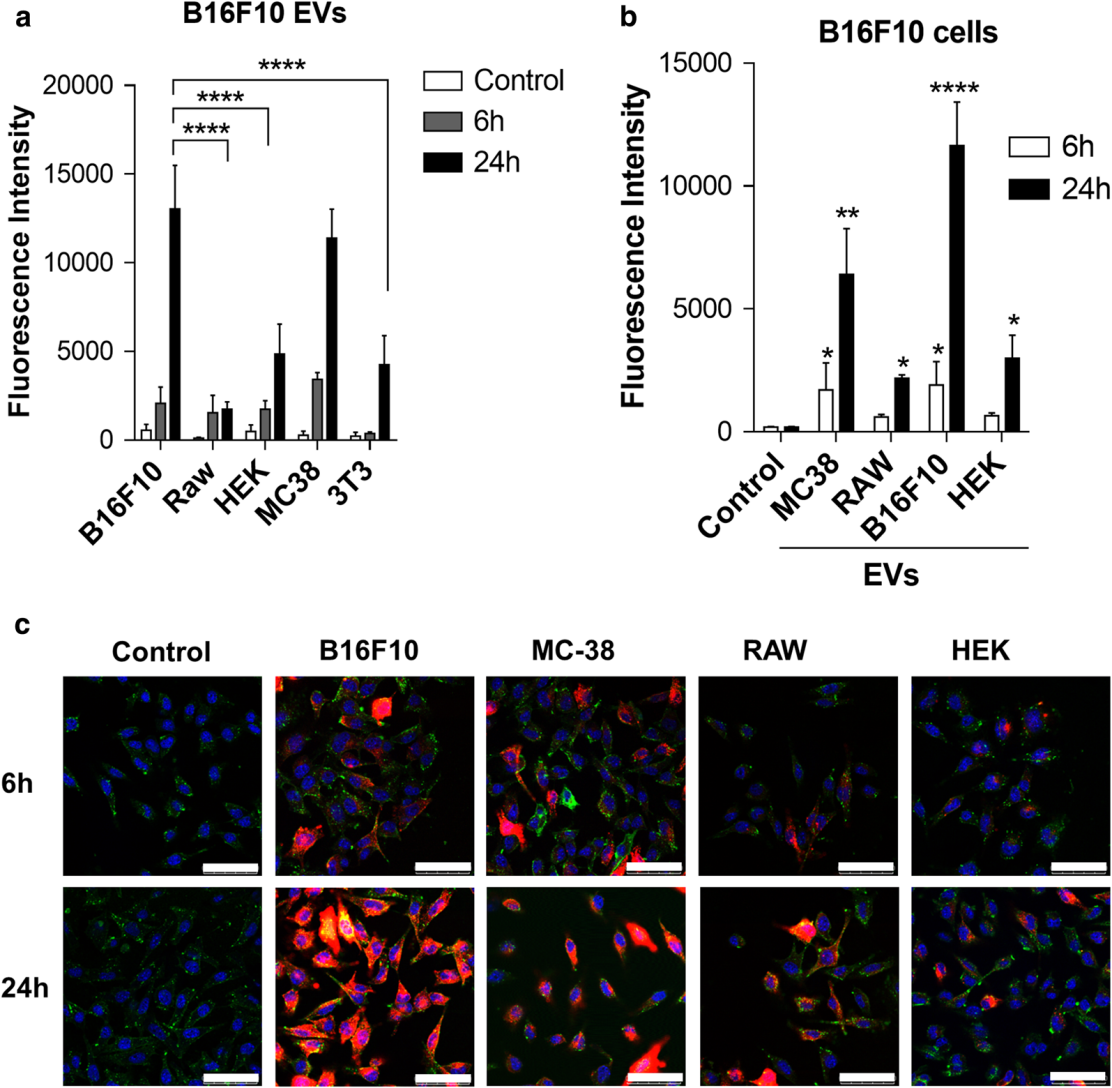
Cell-type specific uptake of B16F10-EVs. **a** B16F10, RAW264, HEK293T, MC38, and NIH3T3 cells were treated with DiD-labeled B16F10 EVs for 6 or 24 h and analyzed by flow cytometry. B16F10 cells were treated with DiD-labeled EVs isolated from either MC-38, RAW264, B16F10 or HEK293T cells and analyzed by **b** flow cytometry and **c** confocal microscopy after 6 or 24 h of incubation. For confocal microscopy, DiD-labeled EVs are shown in red, DAPI in blue and membrane staining with DiO in green. ****P < 0.0001; ***P < 0.001; **P < 0.01; *P < 0.05 (respectively to control, mean ± SEM; n = 4). Scales bar are 50 µm

To determine if uptake of B16F10-EVs was cell-type specific, we performed a multi-culture analysis of five different cell lines and prepared four different types of EVs under similar culture conditions. We applied the membrane tracer DiD, which has been extensively used for EV tracking [39–41]. To ensure that the observed fluorescence was specifically related to the EV adherence/uptake, cells were also incubated with similar concentrations of DiD alone to normalize the results by defining the degree of non-specific binding. As shown in Fig. 2a, the highest uptake, as evaluated by flow cytometry, was observed for B16F10 cells after 24 h of incubation, with notable differences in comparison to the uptake by RAW264, HEK293T and NIH3T3 cells. We also observed that the uptake of MC-38-EVs in B16F10 cells was slightly lower compared to that of B16F10 EVs; however, this difference was not significant, indicating that this uptake may be tumor-specific rather than EV-specific.

Additionally, we compared the uptake of MC-38-EVs, RAW264-EVs, B1610-EVs and HEK293T-EVs by B16F10 cells using confocal microscopy and observed that the uptake of B16F10-EVs was significantly higher than for other EVs, including MC-38-EVs (Fig. 2b). Confocal microscopy of B16F10 cells incubated with the same EVs, revealed similar patterns of uptake, which allows us to conclude that all the EVs (red) were effectively internalized by B16F10 cells (green) (Fig. 2c). Although the appearance of intracellular fluorescence cannot be considered a quantitative, but rather a qualitative measure of EV uptake by cells, our observations comparing the uptake of different EV preparations by B16F10 cells and the uptake of B16F10-EVs by different cells, provides evidence for the preferential uptake of B16F10-derived EVs by B16F10 cells. Other authors have also observed in vitro a cell type specific uptake of EVs in different models, such as ovarian cells [12] and mesenchymal stem cells [42], indicating that the potential of EV specific targeting may not be limited to applications involving cancer cells.

### Synthesis and characterization of AuNP-PEG-FA

**Fig.3:**
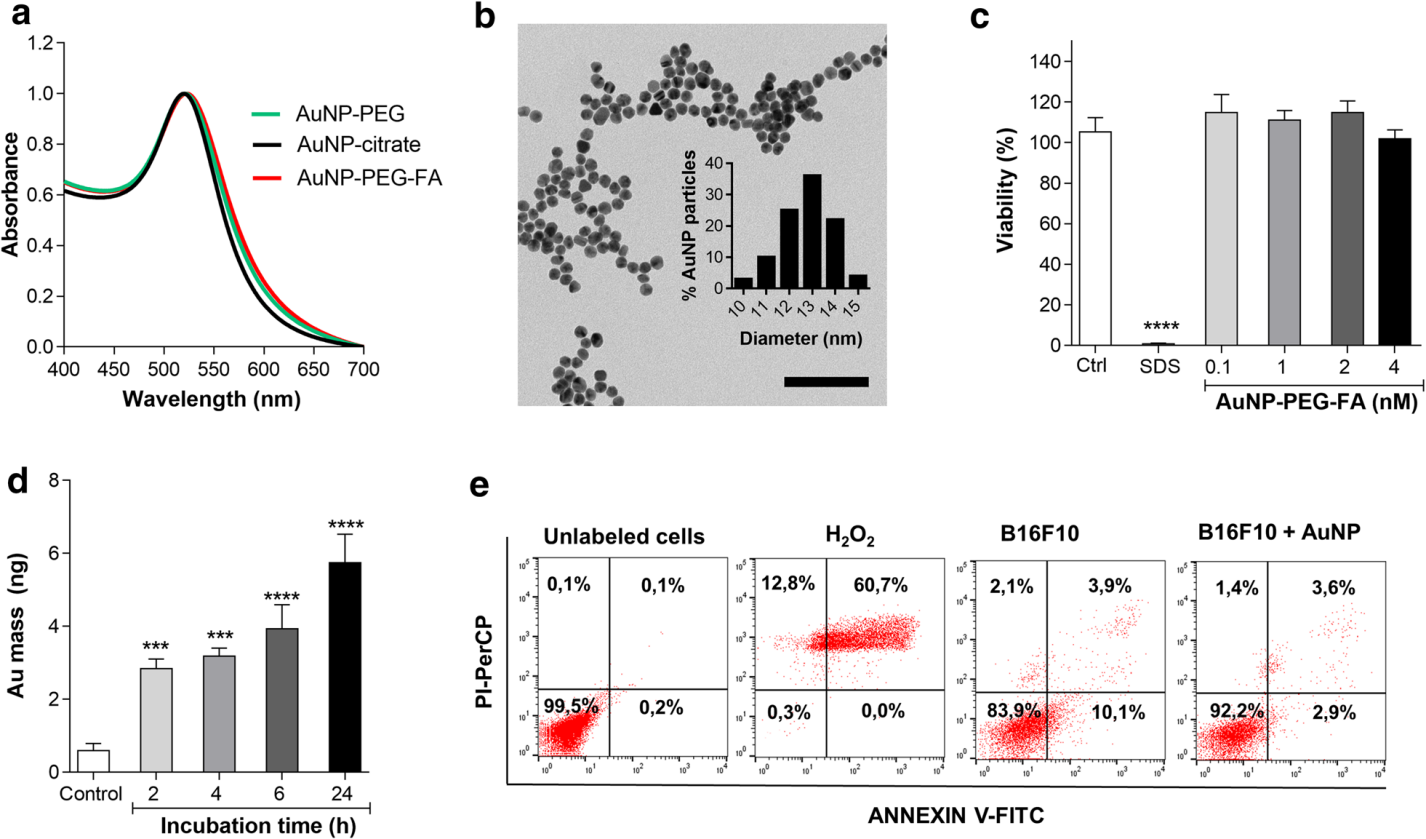
Uptake and cytotoxicity of AuNP-PEG-FA. **a** UV–Vis spectra of AuNP, AuNP-PEG and AuNP-PEG-FA (mean of n = 3). **b** Representative TEM micrograph of AuNP and size distribution obtained from 100 particles. Scale bar is 200 nm. **c** MTS assay of B16F10 cells incubated with different concentrations of AuNP-PEG-FA for 24 h. Ctrl corresponds to cells in the culture medium without AuNPs and SDS corresponds to cells incubated with 2% SDS. **d** Gold content by NAA of B16F10 cells incubated with 1 nM AuNP-PEG-FA for different periods of time. **e** Flow cytometry analysis of confluent B16F10 cells incubated with AuNP-PEG-FA prior to EV isolation. ****P < 0.0001; ***P < 0.001 (respect to control, mean ± SEM; n = 3)

To further study the ability of B16F10 EVs to target tumor cells in vivo and explore their potential in therapy, we sought to incorporate gold nanoparticles into the extracellular vesicles without altering their targeting properties. We used an indirect labeling method to incorporate the therapeutic agent into the EV-producing cells which later secrete the desired compound inside the EVs. As the folic acid receptor is overexpressed in malignant B16F10 cells [43, 44], we used folic acid-conjugated AuNP (AuNP-PEG-FA) to improve the internalization of the AuNP into B16F10 melanoma cells and thereby facilitate their inclusion in EVs. Citrate-coated gold nanoparticles were synthetized as described in methods and subsequently two types of thiol PEGs (methylated and carboxylated) were chemisorbed on their surface. The methylated PEG contributes to the colloidal stabilization [45], while the carboxylated PEG permits conjugation with folic acid [43, 46] following an EDC/NHS protocol. For the nanoparticles, a plasmonic peak of light absorption at 520 nm and an average size of 12 nm with a peak at 13 nm were observed by UV–visible spectroscopy and electron transmission microscopy, respectively (Fig. 3a, b and Additional file 1: Fig. S1a, b). As expected, the conjugation of AuNP with PEG and folic acid resulted in an increase in the hydrodynamic diameter by 9 nm and 14 nm, respectively, as well as a change in the surface potential from a negative value due to citrate (− 201044 mV) to less negative values for PEG (− 16 mV) and for FA (− 31 mV) (Additional file 1: Table S1). The change in the size can be explained by the presence of the molecules on the surface of the nanoparticles, while the changes in zeta potential indicate that at pH = 7, highly negative citrate molecules (three deprotonated carboxyl groups, pKa_1_ = 3.13, pKa_2_ = 4.76, pKa_3_ = 6.4 [43]) were first displaced by PEG-COOH (one deprotonated carboxyl group, pKa = 4.85 [47]) and then by folic acid (two deprotonated carboxyl groups, pKa_1_ = 4.7, pKa_2_ = 6.8 [48]).

### Uptake and cytotoxicity of AuNP-PEG-FA

It has been reported that EV purity and content can be altered by cellular stress and apoptosis [49, 50]. Therefore, we evaluated the effect of increasing concentrations of AuNP-PEG-FA on B16F10 cell viability 24 h post-incubation using the MTS assay. As shown in Fig. 3c, no significant differences were observed in cell viability at the concentrations analyzed. This result was expected, as AuNPs depending on their sizes, shape, charge and surface have been previously described to be non-toxic and highly biocompatible agents [29, 31]. Additionally, to establish if the AuNPs were internalized by B16F10 cells, we measured the gold content in the cells using NAA. Cells were incubated with AuNP-PEG-FA and then irradiated with neutrons. Later, the γ-rays emitted from the samples permitted quantifying the gold content in each sample. Our analysis revealed an increase in gold mass after 2 h of incubation with B16F10 cells (Fig. 3d), equivalent to approximately 4 × 10^3^ AuNP per cell (0.6% of the total). The accumulation of gold in the cells increased after 6 and 24 h of incubation. Similar results were obtained by analyzing the gold nanoparticle presence inside the cells using UV–vis spectrometry, which revealed also an almost threefold higher uptake of AuNP-PEG-FA as compared to AuNP-PEG alone (Additional file 1: Fig. S1c). These results are in agreement with reports by other authors [51, 52] and suggest that AuNP-PEG-FA are effectively taken up by B16F10 cells. We also analyzed cells for evidence of apoptosis or necrosis after AuNP-PEG-FA incubation, as these processes are known to alter the purity and natural targeting of the EVs. As shown in Fig. 3e, we found no evidence for increased levels of Annexin V/PI positive cells after incubation with AuNP-PEG-FA, indicating that our samples were not contaminated by apoptotic bodies.

### Internalization and secretion of AuNP-PEG-FA in B16F10 cells

**Fig. 4:**
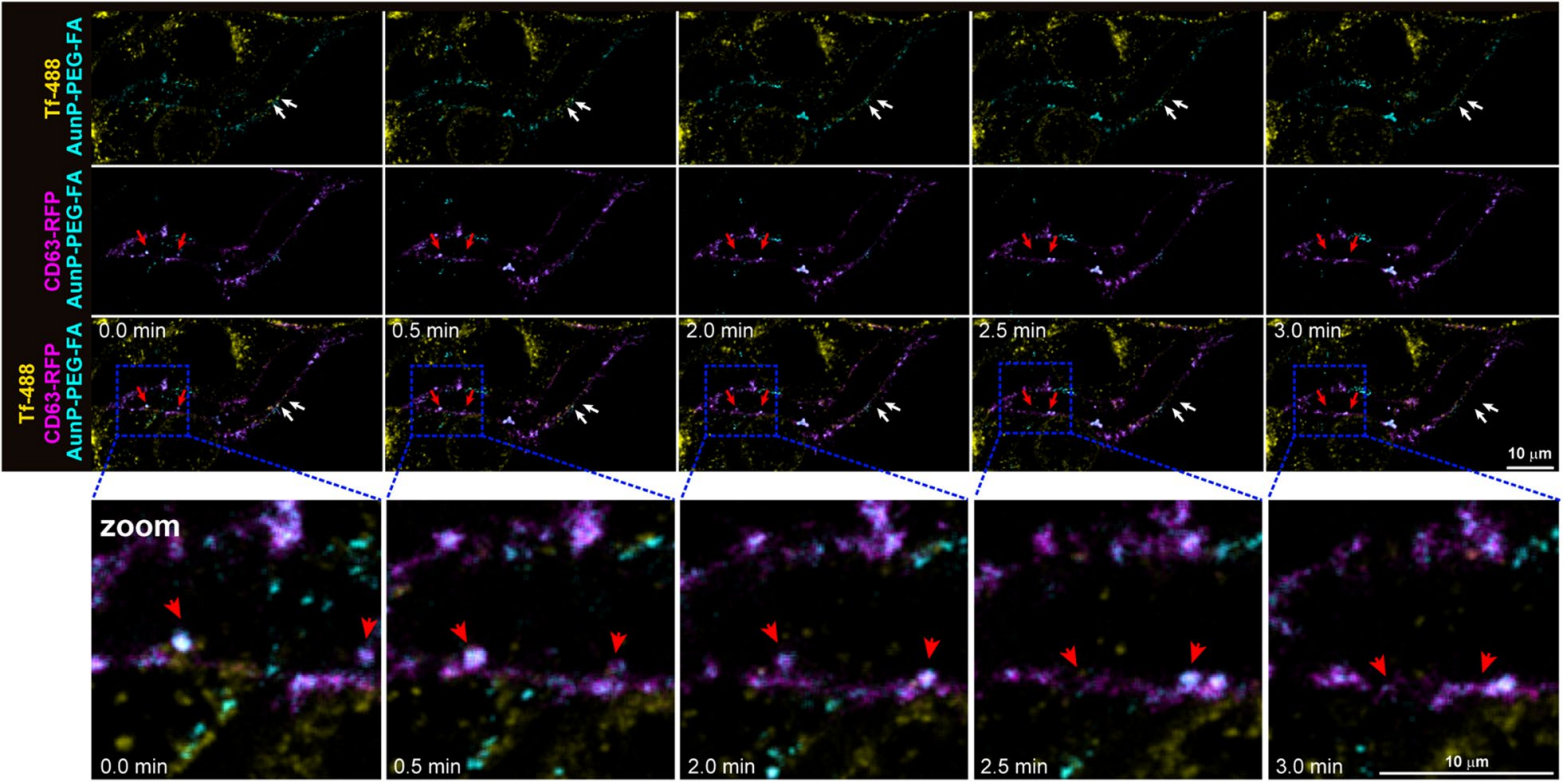
Internalization of AuNP-PEG-FA and secretion from B16F10 cells. B16F10 cells were transfected to express CD63-RFP (magenta) and pulsed with AuNP-PEG-FA (cyan) for 30 min. Then, the cells were incubated with AuNP-PEG-FA in combination with the endocytosis marker Tf-488 (yellow) and imaged in a confocal microscope using time lapse live image acquisition mode. The colocalization of AuNP-PEG-FA with Tf-488 and CD63-RFP is indicated by white and red arrows, respectively. Representative time points are shown. Colocalization of AuNP-PEG-FA with CD63 (dashed blue square) is shown at higher magnification (zoom). Scale bars are 10 μm

Prior to isolation of gold labeled-EVs, we evaluated whether AuNP-PEG-FA were incorporated and secreted by the endocytic/EV pathway in B16F10 cells. B16F10 cells were transfected to express transiently the multivesicular body/EV marker CD63 fused to RFP (CD63-RFP) and incubated with AuNP-PEG-FA for 30 min to promote internalization. Before live image acquisition by confocal microscopy, cells were incubated again with AuNP-PEG-FA and stained with the endocytosis marker transferrin conjugated to Alexa Fluor 488 (Tf-488) (Fig. 4). As transferrin enters the cells through endocytosis and then is trafficked to early endosomes prior to recycling back to the surface [53], the endocytosis of AuNP-PEG-FA (cyan) can be identified by colocalization with Tf-488 (yellow), which is indicated by white arrows (Fig. 4). On the other hand, the secretion of the nanoparticles by the late endosome/EV pathway can be identified by their colocalization with CD63-RFP (red arrows). The combined use of Tf-488 and CD63-RFP, allowed us to track AuNP-PEG-FA nanoparticle internalization and secretion simultaneously (Fig. 4 and Additional file 2: Video S1). Our observations suggest that AuNP-PEG-FA are effectively incorporated by B16F10 cells via endocytosis and then traffic to multivesicular bodies for subsequent secretion as cargos inside EVs.

### Isolation and uptake of double-labeled EVs by B16F10 cells

**Fig. 5:**
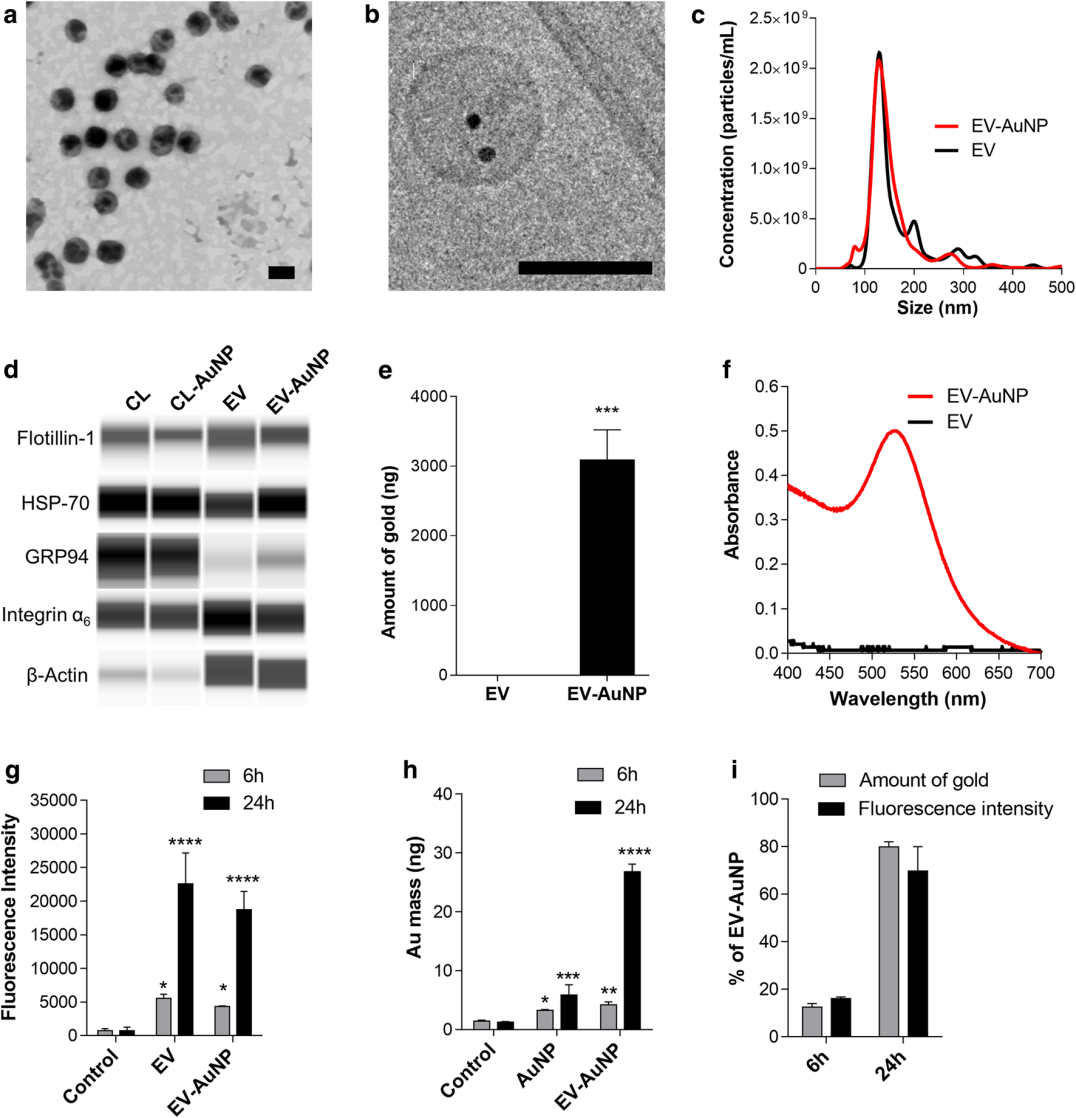
Isolation and uptake of double-labeled EVs by B16F10 cells. Representative **a** TEM and **b** Cryo-TEM micrographs of EV-AuNP; scales bars are both 200 nm. **c** NTA of EV and EV-AuNP. **d** Western blot of cell lysates (CL) and EVs. **e** NAA and **f** UV–Vis spectra of control EVs and EV-AuNP. B16F10 cells were treated with either PBS (control), AuNP-PEG-FA (AuNP), DiR-labeled EVs (EV) or DiR-labeled EV-AuNP (EV-AuNP) for 6 or 24 h. Flow cytometry analysis of **g** DiR fluorescence intensity and **h** NAA of internalized gold after incubation. **i** Percentage of total EV-AuNP internalization determined by fluorescence and gold quantification. ****P < 0.0001; ***P < 0.001; **P < 0.01; *P < 0.05 (respectively to control, mean ± SEM; n = 3)

To show that the AuNPs were effectively secreted as EV-cargos, we collected the supernatants of B16F10 cells after pre-incubation with AuNP-PEG-FA as described in “Methods”. In preliminary assays, AuNPs were retrieved outside the EVs, as evidenced by TEM and DLS (Additional file 1: Fig. S2a, b). Although an additional step of centrifugation permitted removing the AuNPs from the EVs, this step also precipitates EVs containing AuNPs (Additional file 1: Fig. S2c) and, therefore, we chose to add an additional wash step followed by a 24 h incubation with medium to promote release of the EVs. The resulting EVs with AuNPs (EV-AuNP) maintained a size distribution, shape, protein expression and surface charge, similar to control EVs without AuNPs (Fig. 5a–d, Additional file 1: S2d–f and Table S2). EV-AuNP were round in shape (Fig. 5a, b) with a hydrodynamic diameter of 122 ± 4 nm and a zeta potential of − 18 mV, similar to control EVs (Additional file 1: Table S2). NTA analysis revealed a low polydispersity with a mode size of 127 nm and an average concentration of 1 × 10^11^ particles/mL (Fig. 5c). The presence of AuNPs in the vesicles was confirmed by Cryo-TEM (Fig. 5b and Additional file 1: Fig. S2g) together with the presence of a plasmonic band centered at 520 nm (Fig. 5f), which corresponds to the previously observed peak shown in Fig. 3a. The nanoparticles were observed both inside EVs (Fig. 5b) and associated with the outer side of the EV membrane (Additional file 1: Fig. S2g). Total gold content in EV-AuNPs was determined by NAA (Fig. 5e), which together with the NTA analysis allowed us to estimate a total of ~ 1.5 AuNPs per vesicle. Importantly, no unbound gold nanoparticles or peak corresponding to the size of AuNPs was observed by NTA, DLS, TEM or Cryo-TEM (Fig. 5a, c, and Additional file 1: Fig. S2f, g), which indicates that the AuNPs detected were mostly associated with EVs. Given that free AuNPs were not detected by wide-field Cryo-tomography (Additional file 1: Fig. S2g), we conclude that our incubation and isolation method is an efficient strategy to incorporate AuNPs in EVs.

To evaluate if the incorporation of AuNPs affected the uptake of the EVs in B16F10 cells, EV-AuNPs and EV alone were labeled with the fluorescent lipophilic tracer DiR to compare fluorescence with gold-cargo uptake [13, 54–56]. B16F10 cells were incubated with either PBS, AuNP-PEG-FA, DiR-labeled control EVs or double-labeled DiR EV-AuNPs and analyzed by flow cytometry. We observed a fourfold increase in fluorescence intensity from 6 to 24 h, similar to that previously observed in Fig. 2, and no significant differences between control EVs and EV-AuNPs, which indicates that the association of AuNPs did not affect their cell-type specific internalization (Fig. 5g). We then asked if the AuNPs contained in the EVs were also incorporated into tumor cells by measuring the gold content in cells by NAA. As expected, gold analysis of EV-AuNPs revealed an uptake pattern similar to that previously observed by fluorescence analysis (Figs. 2c and 5g, h), which indicates that the EVs and their cargo (AuNP) were effectively incorporated by the cells. Moreover, we compared the results with similar concentrations of AuNP-PEG-FA alone, observing that the incorporation of AuNPs via EVs results in about 4.5 times more efficient internalization by B16F10 cells after 24 h of incubation than direct incubation with the gold nanoparticles alone (Fig. 5h). We calculated that a total of 9 × 10^3^ AuNPs were present per cell after 24 h of direct incubation versus 4 × 10^4^ when AuNPs were not incorporated into EVs (equivalent to 3 × 10^4^ EV-AuNP per cell). To correlate fluorescence results with the total gold content, we calculated the number of EV-AuNPs per cell and observed similar fluorescence/gold ratios (Fig. 5i) confirming that our double labeling strategy was effective. Moreover, our results highlight the potential of B16F10 EVs as drug delivery systems, as they can increase the delivery of gold nanoparticles towards cancer cells. The enhanced uptake of the EVs may be related to their biological structure, which allows them to internalize into cells by many different pathways, such as phagocytosis, micropinocytosis, fusion, endocytosis and receptor-mediated endocytosis [57]. This provides an advantage compared with other drug delivery systems, such as liposomes, for which it has previously been shown that uptake is at least tenfold lower when compared with tumor EVs [58].

### Tracking double-labeled B16F10-EV accumulation in metastatic nodules

**Fig. 6:**
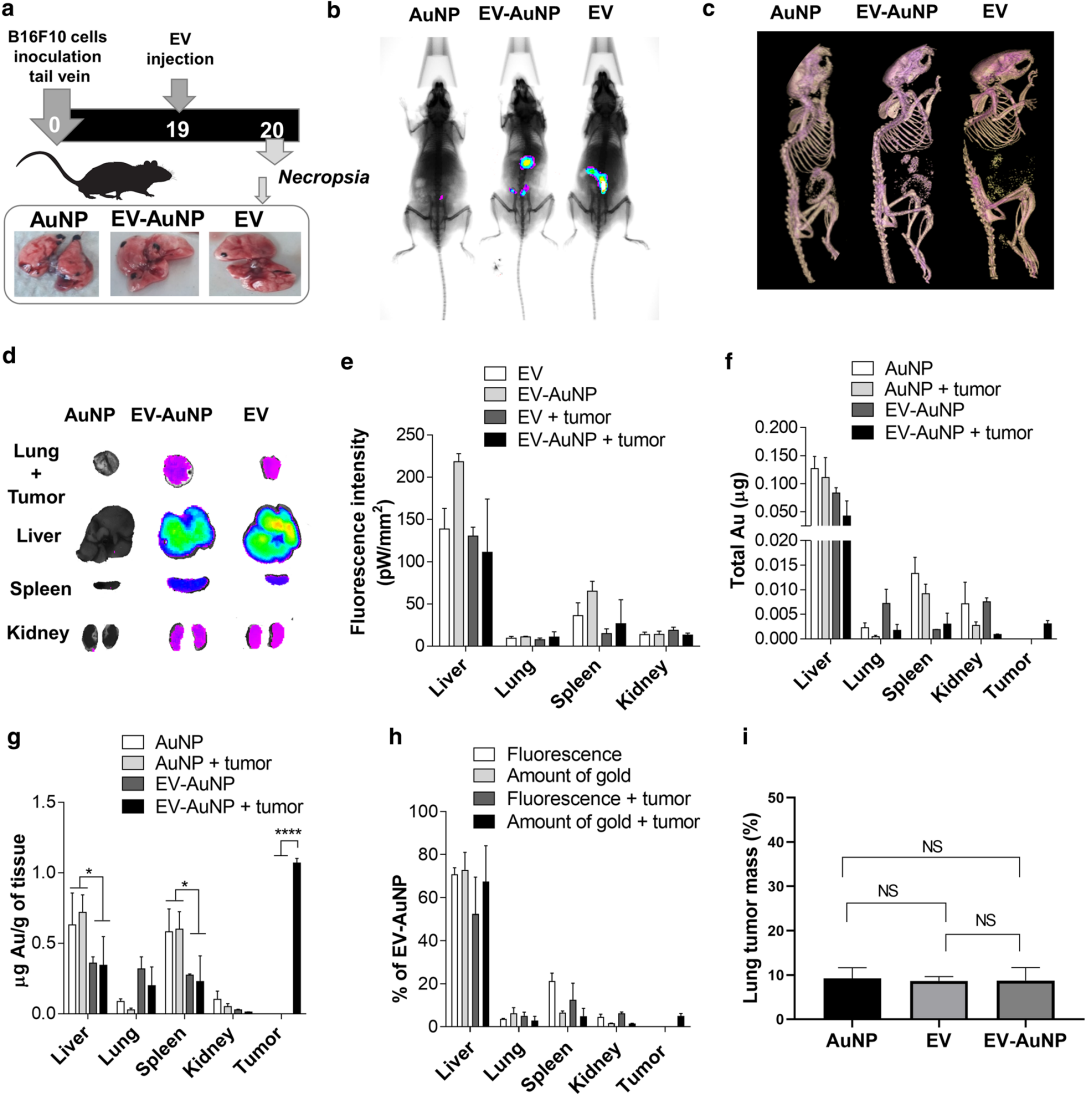
Tracking double labeled B16F10-EV accumulation in metastasis. **a** Experimental design: C57BL/6 mice were injected with 2 × 10^5^ of B16F10 cells to produce lung metastasis and then injected with either DiR-labeled EV (EV), DiR-labeled EV-AuNP (EV-AuNP) or AuNP-PEG-FA (AuNPs). **b** Fluorescence and **c** CT imaging of mice 24 h after injection. **d** Fluorescence imaging of organs and **e** intensity in pW per mm^2^. Gold quantification of tumor nodules and organs expressed as **f** total gold content (μg) and **g** μg/g of tissue. **h** Percentage of total EV-AuNP detected by fluorescence and gold quantification. **i** Determination of tumor mass present in lungs from C57BL/6 mice after injection of AuNP, EV or EV-AuNP. Data are means ± SEM from n ≥ 3 mice per condition. ***P < 0.001; **P < 0.01; *P < 0.05

We selected to use our double labeling strategy to analyze the distribution of B16F10 EVs in metastasis, which has only been reported previously using relative fluorescence analysis and after multiple administrations [38]. Murine B16F10 melanoma cells represent a well-established and versatile model to evaluate metastasis in a pre-clinical setting using syngeneic non-immunosuppressed mice, as the cells selectively metastasize to the lungs of the animals, which permits very precise quantification of metastasis progression around 20 days after injection [59]. B16F10 cells were injected into the tail vein of C57BL/6 mice to produce lung metastasis. Then, after 19 days, the mice were injected with either AuNP-PEG-FA alone, DiR-labeled EV-AuNPs or DiR-labeled EVs and evaluated after 24 h (Fig. 6a). As shown in Fig. 6a, b, whole mouse imaging of fluorescence allowed us to identify the presence of DiD labeled EVs (EV and EV-AuNP) but not AuNP-PEG-FA. Alternatively, by CT imaging we were able to identify AuNP-PEG-FA and EV-AuNPs but not EV-alone. We observed that in both cases, resolution did not suffice to evaluate the distribution in small organs and therefore were not suitable for the imaging of small metastatic nodules in the lungs. This may be attributable to skin autofluorescence, low penetration of light and other effects, such as oxidation, scattering and bleaching, that affect fluorescent imaging [18–20], as well as the presence of gold used for CT imaging [13, 18–20, 32]. Therefore, organs were extracted and analyzed ex vivo. By fluorescence analysis, we observed the highest accumulation of EVs in the liver (Fig. 6d, e), which was to be expected, as the liver is a highly irrigated organ where most of the nanoparticles tend to accumulate, including EVs [13, 60]. Importantly, we observed no significant differences between the distribution of EV-AuNPs and control EVs, indicating that AuNPs did not affect systemic distribution (Fig. 6d, e and Additional file 1: Fig. S3a, b). To analyze distribution towards tumors, the metastatic nodules in lungs were removed and analyzed by NAA. Gold distribution in organs revealed a similar accumulation pattern as was observed by fluorescence, indicating that EVs and their cargo followed similar pathways in the organism (Fig. 6e, f). Interestingly, we observed gold accumulation in tumors of animals treated with EV-AuNPs, but not with AuNP-PEG-FA alone (Fig. 6f) and estimated the presence of a total of 6 × 10^5^ nanoparticles in tumor tissue (5 × 10^5^ EV-AuNP), which corresponds to 0.8% of the injected dose. It is important to mention that the liver mass is about 30 times larger than the micrometastatic tumor nodule mass analyzed and thus the difference in distribution may not accurately reflect the density of EV accumulation. We, therefore, calculated the ratio between gold and tissue mass and observed the highest density of AuNPs in tumors treated with EV-AuNPs and reduced accumulation in liver and spleen compared with AuNP-PEG-FA alone (Fig. 6g). These results indicate that EVs can be used to improve the delivery of AuNPs towards tumors suggesting that these nanosystems are potentially interesting for drug delivery applications and the treatment of metastasis. We analyzed the fluorescence/gold ratios of distribution and observed no significant differences (Fig. 6h), which makes this model interesting for tracking and imaging studies.

It is important to mention that although gold labeling strategies have been previously reported, the possible effects of the gold incorporation on the natural tropism of EVs has not been taken into consideration. We addressed this issue by analyzing control EVs, gold labeled EVs and gold alone using our double labeling strategy. This approach allowed us to determine relevant parameters, such the number of AuNPs per EV, the number of EVs internalized per cell and the number of EVs in tumor tissue, which has been mostly reported as non-quantitative fluorescent intensity units or % of internalization [7, 12, 13, 54].

When compared to other type of EVs, the enhanced uptake, natural tropism and potential for immunotherapy of tumor EVs makes them interesting for drug delivery applications [12, 33]. It is important to note that tumor EVs were also reported to promote tumor formation and metastasis; however, these effects were observed after multiple administrations, such as injections 3 times a week for 3 weeks [38]. We observed that the total tumor/lung mass after a single administration of either AuNPs, EVs or EV-AuNPs (Fig. 6a, i) did not change significantly, indicating that our preparations did not promote tumor growth. We believe that further analysis will be necessary to assess the risk versus benefit of such EVs as delivery systems. Developing strategies to remove the malignant cargo from tumor EVs, while maintaining their targeting capacities, should represent an interesting avenue for future research, as EVs derived from malignant cells are becoming increasingly attractive for drug delivery and immunotherapy purposes [11, 12, 33, 61].

### Detection of gold labeled EVs in small metastatic tissues

**Fig. 7:**
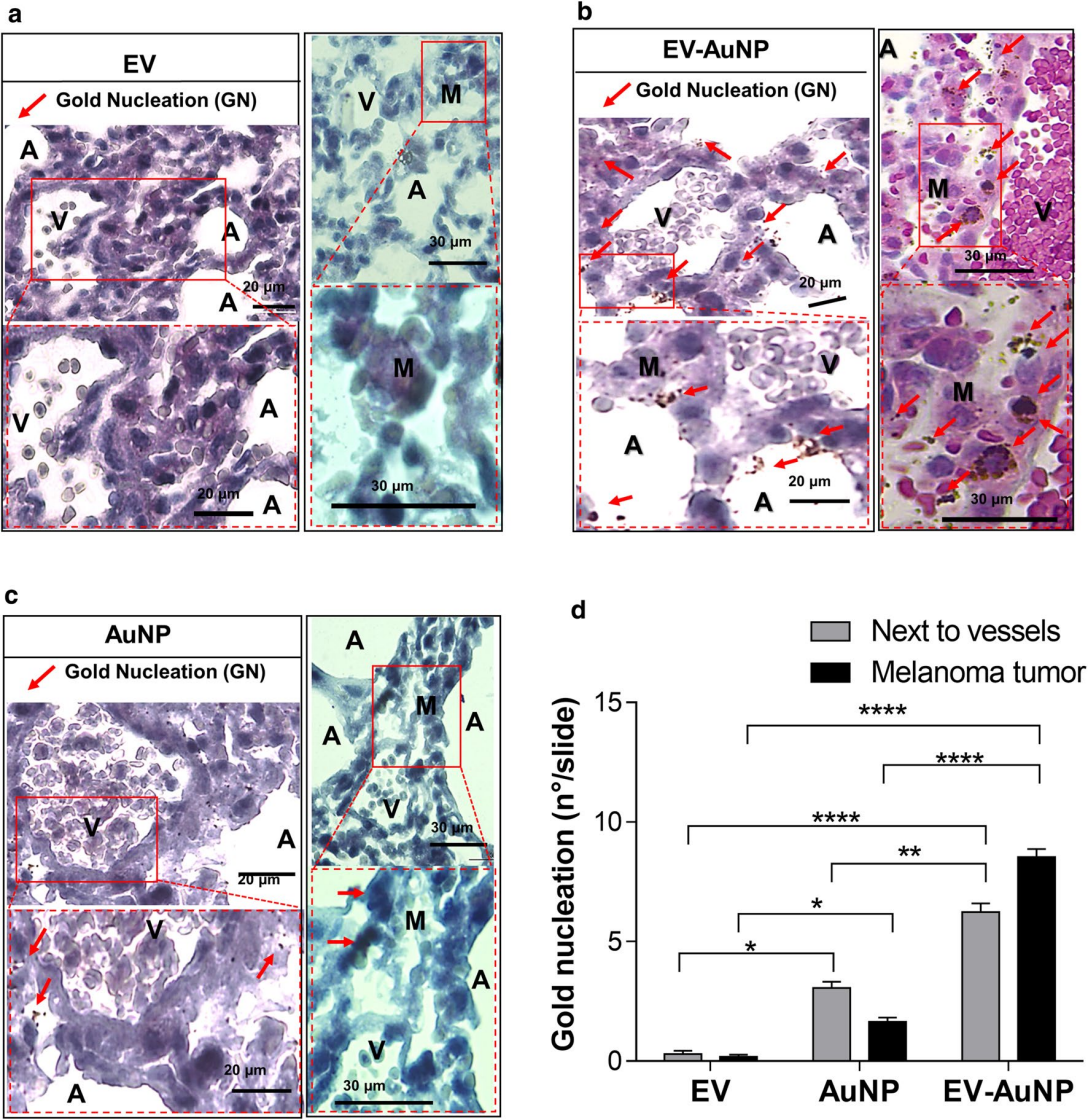
Detection of gold labeled EVs in small metastatic nodules. C57BL/6 mice were injected with 2 × 10^5^ of B16F10 cells to produce lung metastasis and then injected day 19 with either DiR EVs (EV), DiR EV-AuNP (EV-AuNP) or AuNP-PEG-FA (AuNP). On day 20, animals were necropsied, and lungs were analyzed by gold nucleation detection. Histological gold detection in the lungs of mice inoculated with **a** DiR EVs, **b** DiR EV-AuNP and **c** AuNP-PEG-FA. In all cases, dotted squares show magnified images of sections highlighted to the left by red squares, while red arrows indicate the gold nucleation sites (GN). A: Alveolus parenchyma, M: metastatic melanoma nodules and V are the Vessels. **d** Gold nucleation was quantified and graphed for the areas adjacent to the blood vessels and in the lung metastasis nodules. Data are means ± SEM from n = 8 samples per condition. ****P < 0.0001; **P < 0.01; *P < 0.05

To further visualize the distribution of EV-AuNP, lungs were histologically examined to visualize the nanoparticles within the tumor tissue by using a gold nucleation methodology and hematoxylin/eosin staining for contrast. This procedure allows the direct visualization of AuNP distribution in the tumor environment, since each AuNP acts as a nucleus for gold crystal growth to a size that permits visual spot evaluation by optical microscopy. After gold enhancement treatment, it was possible to distinguish between gold accumulation in tumor tissue, alveolus parenchyma and next to the blood vessels (Fig. 7). The highest accumulation was observed in tumor tissue treated with EV-AuNPs, which indicates that the EVs not only reach, but were also taken up by the tumors to deliver their gold nanoparticle-cargo (Fig. 7a, b). In the case of AuNP-PEG-FA, the signal in the tumor nodules was at least five times lower than for EV-AuNP and slightly higher with respect to controls (Fig. 7b–d), which is consistent with our previous results. Interestingly, the highest accumulation of AuNP-PEG-FA was observed close to the blood vessels, which may simply reflect the high degree of vascularization in the lung. It is important to mention that AuNP-PEG-FA are subject to passive targeting through the EPR effect and active targeting do to the presence of the folic acid receptor in tumor cells. The poor degree of initial accumulation in the lung tumors may be because early-stage tumor metastases are usually poorly vascularized which reduces accumulation due to the EPR effect [62]. This would also explain why the accumulation of B16-EVs in tumors (0.8% of injected dose) is lower than what is usually observed in xenograft models that generate well-vascularized tumors. The enhanced uptake of tumor EVs in comparison to AuNPs may then be explained by their natural targeting and adhesion mechanisms. Although the mechanisms of EV tropism towards specific organs are not completely understood, it is known that integrins play an important role in the process. Thus, we evaluated the presence of α6 integrin (Figs. 1g, 5d) and integrin β1 (Additional file 1: Fig. S2h) which are implicated in lung tropism [63] and found that were these integrins were both present in B16F10 cells and B16F10-EVs. This result may explain the increased accumulation in lung tumors of the EVs compared to AuNP-PEG-FA alone, as these integrins likely facilitate accumulation in the lung for subsequent retention in the tumor microenvironment. Therefore, our results support the notion that EV-AuNPs, unlike AuNPs alone, preferentially accumulate in metastatic tumor due to the natural targeting provided by the EVs.

## Conclusions

Overall, the present study identifies the tropism of melanoma B16F10 EVs towards cancer cells and metastatic tumors as well as their potential for drug delivery strategies. We observed that B16F10 cells preferentially take up their own EVs when comparing five different cell lines and four different types of EVs. To further analyze distribution, folic acid-conjugated gold nanoparticles were used to promote cell internalization and trafficking through the endocytic/MVB pathway for subsequent secretion inside the EVs. This approach combined with fluorescence analysis permitted analyzing EV distribution and accumulation by fluorescence/CT imaging, optical microscopy and gold quantification. As our methodology did not alter the natural tropism of the EVs, it can be applied in many other fields to study the trafficking and role of EVs in therapy, diagnosis and the development of diseases. Our quantitative analysis efficiently correlated with fluorescence data and was used to determine the ratio between gold and tissue mass, which we consider a more precise approach to identify the preferential accumulation in organs. Using this strategy, we observed the preferential accumulation of B16F10 EVs in tumor tissue when compared with the other organs. Moreover, using gold-enhanced histological analysis, we determined the precise location of the AuNPs in the tumors and demonstrated the potential of these EVs to increase AuNP delivery towards metastatic tissues. Our findings provide a valuable tool to study the distribution and interaction of EVs in mice and a novel strategy to improve the targeting of gold nanoparticles to metastatic nodules, which could be useful for multiple theranostic applications.

## Methods

### Reagents

If not specified, reagents were purchased from Sigma-Aldrich.

### Synthesis of AuNP

Citrate-coated AuNPs were prepared by citrate reduction of HAuCl_4_. First, the solution of HAuCl_4_ (100 mL, 1 mM) was refluxed for 5–10 min, then a warm (50–60 °C) solution of Na_3_C_6_H_5_O_7_·2H_2_O (10 mL, 38.8 mM) was quickly added. After 30 min of refluxing, the solution of AuNP was filtered through a 0.45 μm polyvinylidene fluoride (PDVF) filter and the pH was adjusted to 7.4. Finally, the colloidal solution of AuNP was stored at 4 °C.

### Preparation of AuNP-PEG-FA

The citrate-coated AuNPs were functionalized with polyethylene glycol (PEG) and folic acid (FA). A solution of AuNPs (10 mL, 5 nM) was incubated with an aqueous solution of HS-PEG-OMe (0.25 mg/50 μL, 5 kDa, Jenkem Technologies) for 10 min at room temperature (RT) and then centrifuged at 16,000×*g* for 60 min to remove the excess of polymer. The nanoparticles were then incubated with an aqueous solution of HS-PEG-COOH (1.5 mg/300 μL, 5 kDa, Jenkem Technologies) for 60 min at RT and centrifuged again. The resulting AuNP-PEG were mixed with 0.2 mg of *N*-(3-dimethylaminopropyl)-*N*′-ethylcarbodiimide hydrochloride (EDC) and 0.5 mg of *N*-hydroxysuccinimide (NHS), dissolved in 0.1 M MES buffer pH 5.5 and sonicated for 15 min at RT. Excess EDC/NHS was removed by centrifugation at 16,000×*g* for 60 min. Next, the pellet was incubated with FA (0.5 mg/500 μL) in PBS buffer overnight at RT. Finally, the solution was centrifuged twice at 16,000×*g* for 60 min and the pellet was resuspended in Milli-Q water.

### Characterization of AuNPs

Plasmon absorbance of AuNP and AuNP-conjugates was determined by UV–visible spectrophotometry in a Perkin Elmer Lambda 25 UV/VIS Spectrometer. Additionally, hydrodynamic diameter and zeta potential of the nanoparticles were measured by dynamic light scattering (DLS) and laser doppler micro-electrophoresis respectively, with a Zetasizer Nano-ZS (Malvern). Finally, the size and morphology of the AuNP were observed by transmission electron microscopy (TEM) in a Hitachi HT7700 microscope.

### Calculation of AuNP concentration

The total content of gold in samples was determined by neutron activation analysis (NAA) at the Comisión Chilena de Energía Nuclear (CCHEN). The samples were lyophilized, sealed by friction welding and exposed for 17 h to a neutron flux of 0.25–1.3 × 10^13^ n/cm^2^s with a power source of 5 mW using a RECH-1 reactor at CCHEN. This procedure triggers the conversion of ^197^Au to ^198^Au. After 7–12 days of decay, the γ-rays emitted by the samples were measured using a germanium detector coupled to a PC-based multichannel γ-ray spectrometer. The γ-spectra were analyzed using the software SAMPO90 Canberra. Gold standards were run with the experimental samples to standardize a library of gold element data, from which the amount of gold present in the unknown samples was calculated. Given the fact that the elemental composition of the sample can influence detection limits by neutron activation, background levels were determined by irradiating untreated (control) tissue samples of a similar size and composition.

### Cell viability assays

The effect of AuNP-PEG-FA on cell viability was evaluated by the 3-(4,5-dimethylthiazol-2-yl)-5-(3-carboxymethoxyphenyl)-2-(4-sulfophenyl)-2*H*-tetrazolium (MTS) assay (Promega). Briefly, 1 × 10^4^ B16F10 cells were seeded in 96-well plates and incubated at 37 °C, 5% CO_2_. After 24 h, the medium was replaced with 100 μL of increasing concentrations of AuNP-PEG-FA in RPMI medium and incubated for another 24 h. The cell viability was measured (in quintuplicate) in three independent experiments using the MTS assay according to the manufacturer’s protocol. Cell death by apoptosis or necrosis was evaluated before EV isolation by flow cytometry. Briefly, B16F10 cells were grown to 50% confluency and incubated with AuNP-PEG-FA (1 nM) for 6 h at 37 °C, 5% CO_2_. Non-incorporated nanoparticles were discarded by washing 3 times with PBS and the medium was replaced with RPMI supplemented with 10% of EV-free serum prepared as previously described [64]. After 24 h, cells were harvested and marked with FITC Annexin V and propidium iodide (PI) using a cell death/apoptosis kit (Invitrogen), according to the manufacturer's protocol and analyzed by flow cytometry with a FACScanto A (BD Biosciences).

### AuNP-PEG-FA cell uptake

B16F10 cells were plated in 24-well plates at a density of 6 × 10^4^ cells per well and then treated with 0.5 nM AuNP-PEG-FA and incubated at 2, 4, 6 or 24 h prior harvest at 37 °C, 5% CO_2_. Incubations were initiated such that all samples could be collected at the same time to ensure that similar amount of cells were present in all cases. After the incubation, cells were washed 3 times with PBS to remove non-incorporated nanoparticles and then harvested and lyophilized. The gold content was determined by NAA as described above. For confocal analysis, B16F10 cells were transfected using LT-Transit (Mirus) with the plasmid pCT-CD63-RFP (System Biosciences), which results in transient expression of the multivesicular/late endosome marker CD63 fused to RFP. Cells were then seeded on glass-bottom Petri dishes (MatTek), allowed to grow for 46 h and pulsed with 1 nM AuNP-PEG-FA for 30 min at 37 °C in phenol red-free DMEM medium supplemented with 10 mM HEPES and 5% FBS. Cells were then incubated again with 1 nM AuNP-PEG-FA in combination with 5 nM of the endocytosis marker Transferrin conjugated to Alexa Fluor 488 (Tf-488, Thermofisher). Finally, cells were transferred to a SP8 Leica spectral confocal microscope equipped with a temperature control chamber (Okolab) for time-lapse live image acquisition. Images were acquired every 30 s for 10–20 min at 37 °C using the 488 nm, 561 nm and 633 nm lasers for the detection of Tf-488, CD63-RFP and AuNP-PEG-FA, respectively.

### EV isolation

To isolate EVs, B16F10, HEK-293T, RAW264, MC-38 and NIH3T3 cells were grown to a density of 18.4 × 10^6^ in 225 cm^2^ flasks in EV-depleted culture medium (culture information of individual cells available in Additional file 1). After reaching 80–90% confluency, the medium was collected and centrifuged at 300×*g* for 10 min, followed by 2000×*g* for 30 min and 16,000×*g* for 30 min. The supernatant was filtered through 0.22 μm membranes and incubated with an EV precipitation buffer (Cellgs^®^) overnight at 4 °C. The mixture was then centrifuged at 16,000×*g* for 60 min and resuspended in 100 μL of PBS before isolation using Exo-spin columns (Cellgs^®^) according to the manufacturer's protocol. For the isolation of EVs loaded with AuNPs (EV-AuNP), B16F10 cells were grown to 50% confluency and incubated with AuNP-PEG-FA (1 nM) for 6 h at 37 °C, 5% CO_2_ to promote gold internalization. Non-incorporated nanoparticles were discarded by washing 3 times with PBS and the medium was replaced with RPMI supplemented with 10% of EV-free serum. Cells were then incubated for an additional 24 h to promote release of EV-AuNP. The resulting medium was collected and centrifuged at 300×*g* for 10 min followed by 2000×*g* for 30 min and filtered through 0.22 μm membranes. EVs containing AuNPs were pelleted by centrifugation at 16,000×*g* for 60 min, resuspended in PBS and incubated with an EV precipitation buffer overnight at 4 °C. The precipitate was then centrifuged at 16,000×*g* for 60 min and resuspended in 100 μL of PBS before the purification using the Exo-spin columns according to the manufacturer's protocol.

### Characterization of EV preparations

Hydrodynamic diameter and surface charge of EVs were analyzed by dynamic light scattering (DLS) and laser Doppler micro-electrophoresis (LDA), respectively, with a Zetasizer Nano-ZS (Malvern). EVs were diluted 100-fold in PBS and then loaded on a disposable polycarbonate capillary cell (DTS 1061, Malvern) maintained at precisely 25 °C. Additionally, purified EVs were diluted in PBS and analyzed by nanoparticle tracking analysis (NTA) to determine size distribution and particle concentration with a Nanosight^®^ NS300 (Malvern). The parameters used for EV detection were a camera level of 9 and automatic functions for all post-acquisition settings except for the detection threshold, which was fixed at 3. The size and morphology of EVs were determined by TEM. Briefly, 5 µL of the samples were dropped on a copper grid and allowed to interact for 2 min. Subsequently, the grid was washed with a drop of water for one min, then stained with a drop of 0.5% phosphotungstic acid for 30 s, washed again and left to dry overnight. Finally, samples were observed by TEM in a FEI Inspect F50. For Cryo-TEM analysis, 5 µL of either EV or EV-AuNP were pipetted onto the carbon surface of a glow-discharged Lacey Carbon 300 mesh copper grid (Ted Pella, USA). The cryo-immobilization was performed in a Vitrobot Mark III (FEI Company, Eindhoven, Netherlands) by plunge freezing in liquid ethane. The sample was kept at 100% humidity and the excess of liquid was automatically blotted with filter paper. Vitrified samples were stored in liquid nitrogen until further analysis by the cryo-electron microscopy. Plunge-frozen samples were transferred to a Tecnai F20 EM (FEI, Eindhoven, The Netherlands) using a cryo-holder (Gatan, Pleasanton, USA). The sample was examined at 200 kV, at temperatures ranging from − 179 to − 170 °C and using low-dose imaging conditions. Low-dose images were recorded at 4096 × 4096 pixel resolution with a CCD Eagle camera (FEI, Eindhoven, The Netherlands). To determine the total protein content in EV isolates the MicroBCA protein assay kit (Thermo Fisher) was used. To detect EV-proteins, capillary electrophoresis was performed using the Protein Simple Wes according to the manufacturer’s instructions. Briefly, samples (Cell lysates and EVs) were lysed with a buffer containing 20 mM HEPES and 0.5 mM PMSF (phenylmethylsulfonyl fluoride), as well as the phosphatase inhibitor ortho-vanadate (OVA, 50 mM in PBS 1×). 0.8 µg/µL of lysed proteins were then mixed with the provided SDS/DTT mix, boiled at 95 °C for 5 min and loaded into a prefilled microwell plate. Primary antibodies, blocking buffer, luminol/peroxidase, HRP streptavidin, and secondary anti-rabbit antibody provided by the manufacturer (anti rabbit detection module, protein simple) were then subsequently loaded into the microplate and spun for 5 min at 300×*g*. The plate was then placed into the instrument for electrophoretic separation using 25-capillary cartridges for 12–230 kDa protein separation (SM-W004). Anti-Grp94 (Sigma, 1:10), anti-Flotillin-1 (Cell signal, 1:10), anti-HSC70 (Cell signal, 1:100), anti-α6-integrin (Cell signal 1:10), anti-β1-integrin (Cell signal, 1:20) or anti-β-actin (BioLegend, 1:50) antibodies were used as primary antibodies for the assays. Chemiluminescent bands were digitally generated and analyzed using the Compass software (ProteinSimple). The gold content in EV-AuNP was determined by evaluating plasmon absorbance using a UV–visible spectrophotometry and NAA as described above.

### Preparation of DiR and DiD-labeled EVs

Freshly isolated EVs were incubated with either 1 μM of the fluorescent lipophilic tracer DiR (D12731, LifeTechnologies) or 1 μM of DiD (D7757, LifeTechnologies) at 4 °C for 30 min. 100 μL of each sample were placed on top of a size exclusion column (Exo-spin®) and centrifuged at 50×*g* for 1 min. 200 µL of PBS were then placed on top of the column and the EVs were obtained after centrifugation at 50×*g* for 1 min.

### Uptake of EVs

B16F10 cells were plated in 24-well plates at a density of 6 × 10^4^ cells per well and then incubated with either DiD-labeled B16F10, MC38, RAW264, HEK293T EVs (10 µg of total protein) for 6 or 24 h. In parallel, B16F10, RAW264, HEK293T, MC38 and NIH3T3 cells were seeded at the same density and incubated with 10 μg of B16F10 EVs or DiD alone for 6 or 24 h. Then, cells were washed twice with PBS and a total of 10,000 cells per condition were analyzed by flow cytometry using a BD LSR-II. Mean fluorescence intensity of DiD alone was used to normalize results between different cell lines. For confocal microscopy, B16F10 cells were plated at the same density and treated with 10 µg of either DiD-labeled B16F10, RAW264, MC-38 or HEK293T EVs. After 24 h of incubation, cells were stained with DAPI for nuclear staining and DiO for membrane staining and then analyzed using a confocal microscope, model Leica SP8. Images were acquired using a 405 laser for DAPI, 488 nm for membrane staining (DiO) and 633 nm for DiD-labeled EVs. For the uptake of gold-labeled EVs, B16F10 cells were plated in 24-well plates at similar density per well and then incubated with either DiR-labeled B16F10 EVs (10 µg of total protein), DiR-labeled B16F10 EV-AuNP (10 µg of total protein, 0.5 nM gold), AuNP-PEG-FA (0.5 nM gold) or PBS for 6 or 24 h. Then, cells were washed twice with PBS and a total of 10,000 cells per condition were analyzed by flow cytometry (FACScanto). Additionally, quantitative analysis of EV-AuNP uptake by B16F10 cells was determined by measuring the incorporated gold content by NAA analysis as described above.

### Animal experiments

Animals C57BL/6 mice were housed in polycarbonate cages placed in a ventilated, temperature-controlled room at 20 °C and 10% relative humidity, under a 12-h light/dark cycle. Standard rat chow and filtered water were available ad libitum. The animals were acclimatized to this environment for at least 1 day prior to treatment.

### Biodistribution assay

8-week-old C57BL/6 mice were injected intravenously via the tail vein with 100 μL of either DiR-labeled EVs, EV-AuNP (0.5 nM, 10 µg of protein) or AuNP-PEG-FA (0.5 nM). After 6 or 24 h mice were sacrificed, and fluorescence images of animals and organs were captured by In-Vivo FX PRO (Bruker) imaging. Noninvasive imaging was performed using a small-animal CT system (nanoSPECT/CT^®^, Bioscan, Washington, DC). Additionally, for gold analysis, the organs were lyophilized and analyzed by NAA as described above.

### Metastasis assay and histological analysis

8-week-old C57BL/6 mice were injected intravenously with 2 × 10^5^ B16F10 cells (in 500 μL of physiological saline solution, 0.9% NaCl). After 19 days, mice were injected with 100 μL of either DiR-labeled EVs (10 μg), EV-AuNP (0.5 nM, 10 μg of protein), AuNP-PEG-FA (0.5 nM) or PBS. After another 24 h the animals were necropsied and organs were visualized using the In-Vivo FX PRO imaging system (Bruker). Black tissue was separated from the rest of the lung and weighed. Metastasis was expressed as black tissue mass/total lung mass in percent (%) post-fixation, as previously described [59]. Organs and a section of metastatic lung tumor nodules were then lyophilized for gold NAA analysis as described above. In parallel, a section of extracted lungs was fixed (PFA 4%) for 48 h at 4 °C, embedded in paraffin and sectioned at 5 μM using a microtome for gold nucleation analysis as previously described [65]. Briefly, tissues were deparaffinized, hydrated and treated with Retrieval solution (DAKO, Rune Linding) at 100 °C for 20 min. The slices were then washed with PBS, 50 nM glycine, 0.1% gelatin and water. Tissues were then allowed to react for 15 min with 200 μL of Gold Enhanced™ LM (Nanoprobes) and rinsed with water to stop gold nucleation. Finally, samples were stained with Contrast BLUE solution and mounted with DAKO mounting solution. These experiments were repeated three times. To observe gold nucleation, at least five digital images were obtained per sample by light microscopy using 40–100× magnification and identical camera ettings.

### Statistical analysis

Statistical analyses of the data were performed using Prism 6.0 (GraphPad Software Inc.) by applying the nonparametric test ANOVA followed by the Tukey post-test for all p-values unless specified in the text. All results are expressed as mean ± SEM.

## Supplementary information

**Additional file 1.** Cell culture condition.** Tables S1,S2**,** Figures S1–S3.** Data summarizing characterization of AuNP, AuNP-PEG, AuNP-PEG-FA, B16F10 EVs and EV-AuNP by DLS, Laser Doppler Anemometry, TEM, Cryo-TEM and western blot. Uptake of AuNP-PEG and AuNP-PEG-FA in B16F10 cells. Distribution of control EVs, AuNP-PEG-FA and EV-AuNP in mice without tumors by fluorescence imaging.

**Additional file 2.** Endocytosis and secretion of AuNP-PEG-FA by B16F10 cells.

## Data Availability

The datasets used and/or analysed during the current study are available from the corresponding author on reasonable request.

## Abbreviations

Exo: B16F10 exosomes
MVB: multivesicular bodies
AuNP: gold nanoparticles
EVs: extracellular vesicles
EV-AuNP: B16F10 EVs with association of AuNP
PEG: polyethylene glycol
FA: folic acid
AuNP-PEG-FA: AuNP conjugated with PEG and FA
EPR: enhanced permeability and retention
EDC: *N*-(3-dimethylaminopropyl)-*N*′-ethylcarbodiimide hydrochloride
NHS: *N*-hydroxysuccinimide
SERS: surface enhanced Raman spectroscopy
CT: computer tomography
NTA: nanoparticle tracking analysis
GN: gold nucleation
TEM: transmission electron microscopy
Tf: transferrin
DLS: dynamic light scattering
NAA: neutron activation analysis
PI: propidium iodode
PTT: photothermal therapy
CCHEN: Comision Chilena de Energía Nuclear
MTS: 3-(4,5-dimethylthiazol-2-yl)-5-(3-carboxymethoxyphenyl)-2-(4-sulfophenyl)-2*H*-tetrazolium
