# Supplementary material for: Gold nanoparticle based double-labeling of melanoma extracellular vesicles to determine the specificity of uptake by cells and preferential accumulation in small metastatic lung tumors
Source: J Nanobiotechnology. 2020 Jan 23;18:20. doi: 10.1186/s12951-020-0573-0 (PMC6979068; doi:10.1186/s12951-020-0573-0)
Supplement: Supplementary file 1 — Additional file 1. Cell culture condition. Tables S1,S2, Figures S1–S3. Data summarizing characterization of AuNP, AuNP-PEG, AuNP-PEG-FA, B16F10 EVs and EV-AuNP by DLS, Laser Doppler Anemometry, TEM, Cryo-TEM and western blot. Uptake of AuNP-PEG and AuNP-PEG-FA in B16F10 cells. Distribution of control EVs, AuNP-PEG-FA and EV-AuNP in mice without tumors by fluorescence imaging. [file 12951_2020_573_MOESM1_ESM.pdf]

Electronic supporting information

Cell culturing conditions.

Metastatic murine melanoma cells (B16F10 ATCC) and colon carcinoma cell lines (MC-38) were cultured in cultured in RPMI 1640 (GIBCO) supplemented with 10% FBS (Biological Industries) and antibiotics (100 U/ml penicillin, 100 mg/ml streptomycin). Murine macrophages (RAW264 ATCC), murine fibroblast (NIH3T3, ATCC) and human embryonic kidney cells (HEK293T ATCC) were cultured in Dulbecco's Modified Eagle Medium with GlutaMAXTM (DMEM, GIBCO) supplemented with 10% FBS (Biological Industries) and antibiotics (100 U/ml penicillin, 100 mg/ml streptomycin. Cells were maintained at 37 °C under 5% CO2 pressure

Surface modification	Dh (nm) ± SD	PDI	pZ (mV)
Citrate	21 ± 0.3	0.26	-44 ± 2
PEG	29 ± 0.5	0.28	-16 ± 2
PEG-FA	36 ± 0.4	0.36	-31 ± 4

Table S1. Hydrodynamic diameter (Dh) by dynamic light scattering and zeta potential (pZ) by laser doppler micro electrophoresis of AuNP, AuNP-PEG and AuNP-PEG-FA.

	Dh (nm) ±SD	pZ (mV) ± SD
Control EVs	127 ± 3	-17 ± 2,0
EV-AuNP	122 ± 4,0	-18 ± 1,0

Table S2. Hydrodynamic diameter and zeta potential of EVs obtained from DLS and Laser Doppler Anemometry

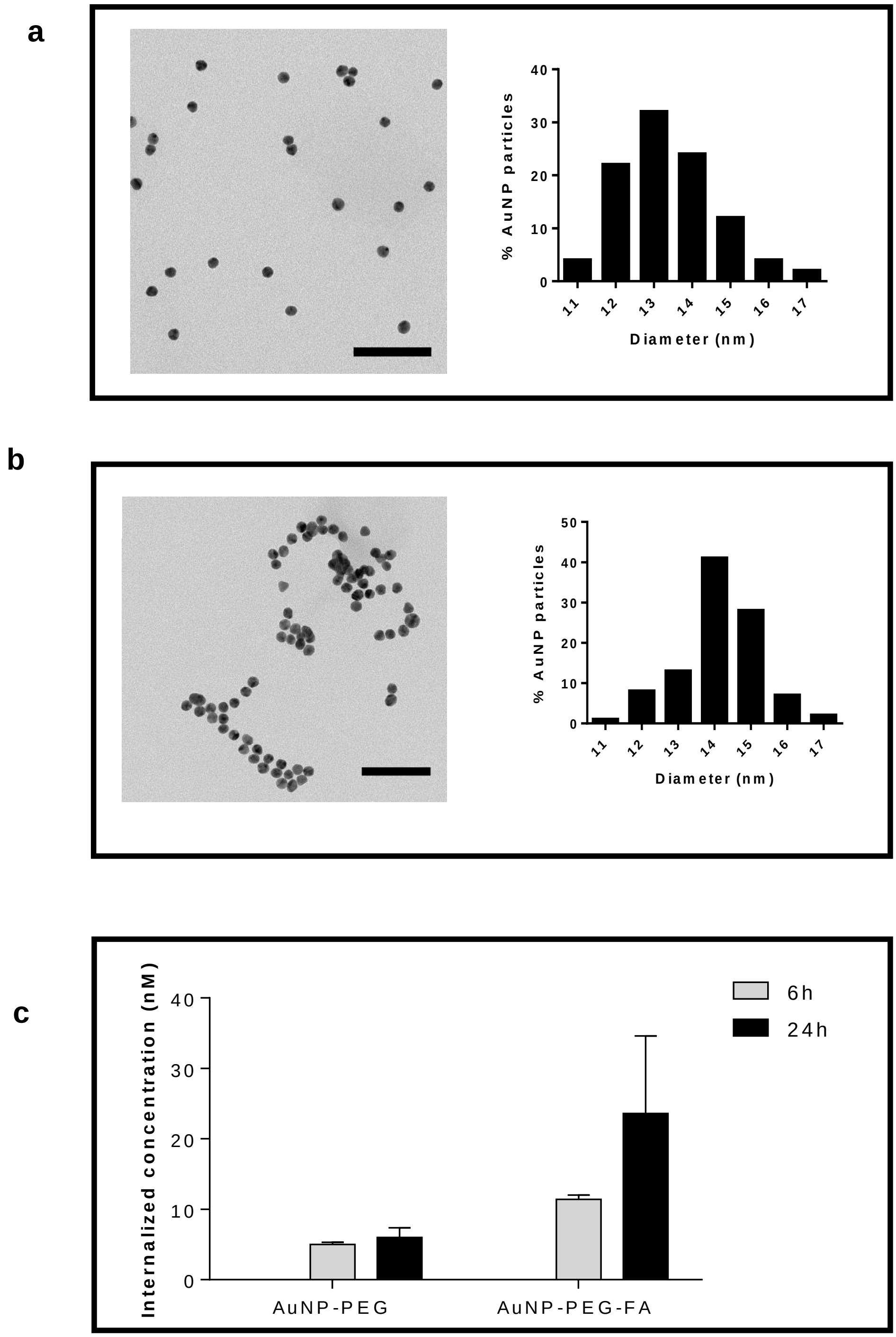

Figure S1. Representative TEM Micrograph of (a) AuNP-PEG-COOH and (b) AuNP-PEG-FA . Histograms are obtained from 100 particles and scale bar is 200 nm. (c) B16F10 cells were incubated with either AuNP-PEG and AuNP-PEG-FA for 6 or 24 hours. Total gold content was analyzed by UV-vis spectroscopy and correlated with the internalized concentration

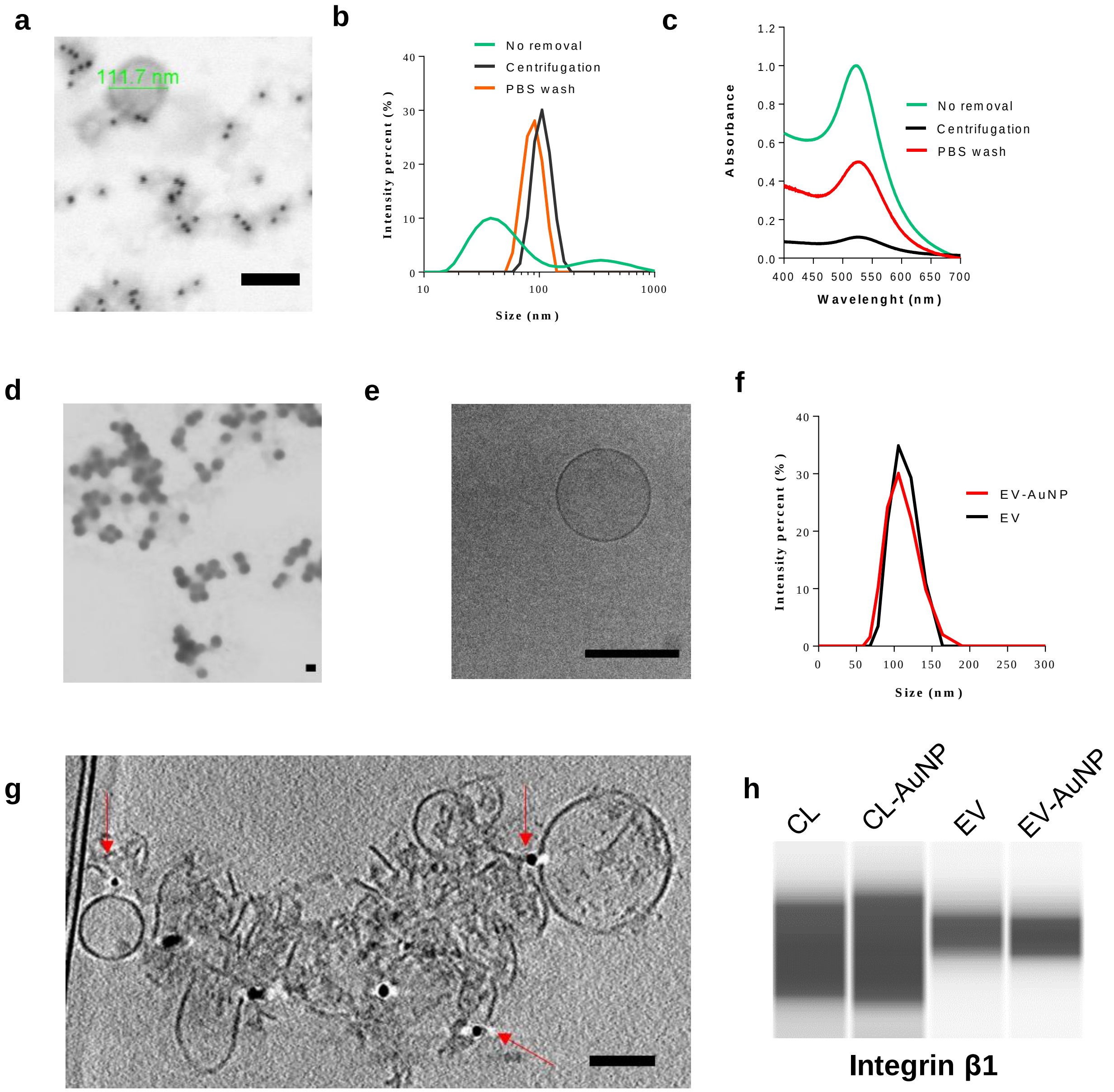

Figure S2. (a) TEM images of particles obtained from AuNP-treated B16F10 cells after normal isolation protocol and (b) DLS after additional centrifugation (16,000 x g, 1h) or wash step. (c) UV-vis spectroscopy of resulting particles, PBS wash correspond to EV-AuNP chosen for isolation. (d) TEM and (e) Cryo-TEM of control EVs (f) DLS of EV-AuNP and control EVs. (g) Cryo-TEM of Exo-AuNPs in which gold nanoparticles (red arrows) can be observed in outer membrane of the EVs. Scale bars are 100 nm (figure extracted from a Cryo-tomography). (h) Western blot of cell lysates (CL), Evs and EV-AuNPs

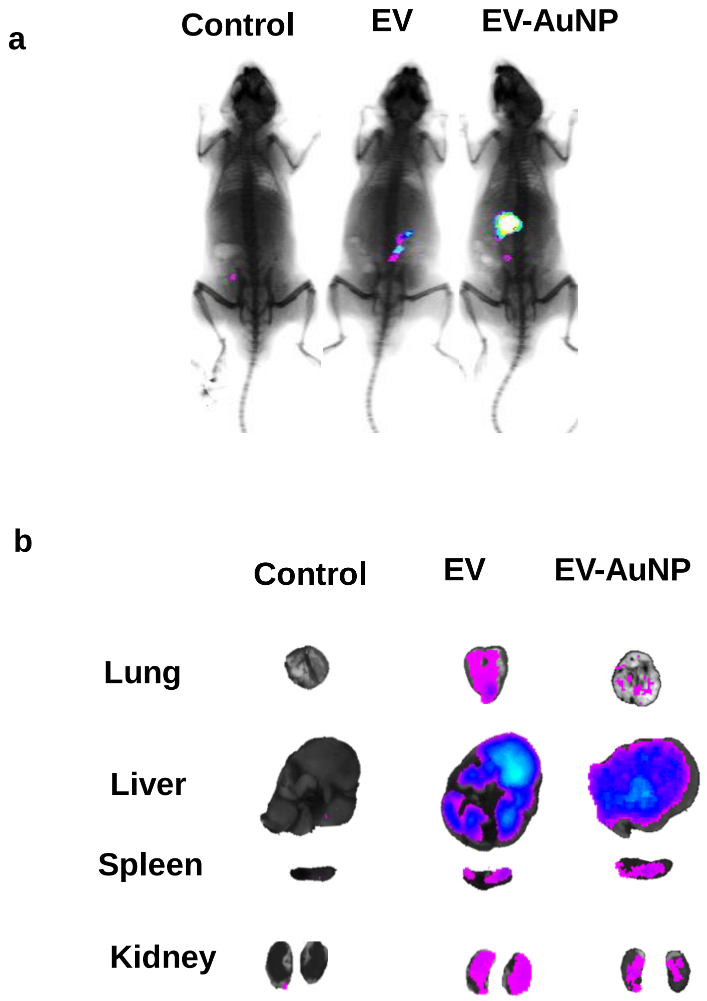

Figure S3. C57BL/6 mice were injected by tail vein with 100 μL of either AuNP-PEG-FA, DIR EVs or DIR EV-AuNP. Fluorescence imaging of (a) mice and (b) organs 24 hours after treatment
